# Measuring Semantic Relatedness between Flickr Images: From a Social Tag Based View

**DOI:** 10.1155/2014/758089

**Published:** 2014-02-23

**Authors:** Zheng Xu, Xiangfeng Luo, Yunhuai Liu, Lin Mei, Chuanping Hu

**Affiliations:** ^1^The Third Research Institute of Ministry of Public Security, 339 Bisheng Road, Shanghai 201142, China; ^2^Shanghai University, 149 Yanchang Road, Shanghai 200472, China

## Abstract

Relatedness measurement between multimedia such as images and videos plays an important role in computer vision, which is a base for many multimedia related applications including clustering, searching, recommendation, and annotation. Recently, with the explosion of social media, users can upload media data and annotate content with descriptive tags. In this paper, we aim at measuring the semantic relatedness of Flickr images. Firstly, four information theory based functions are used to measure the semantic relatedness of tags. Secondly, the integration of tags pair based on bipartite graph is proposed to remove the noise and redundancy. Thirdly, the order information of tags is added to measure the semantic relatedness, which emphasizes the tags with high positions. The data sets including 1000 images from Flickr are used to evaluate the proposed method. Two data mining tasks including clustering and searching are performed by the proposed method, which shows the effectiveness and robustness of the proposed method. Moreover, some applications such as searching and faceted exploration are introduced using the proposed method, which shows that the proposed method has broad prospects on web based tasks.

## 1. Introduction

Relatedness measurement especially similarity between multimedia such as images and videos plays an important role in computer vision. The image similarity is a base for many multimedia related applications including image clustering [1], searching [2, 3], recommendation [4], and annotation [5]. The relatedness problem is relevant to two aspects: images representation and relatedness measurement. The former aspect needs an appropriate model to reserve the related information of an image. The latter aspect requires an effect method to compute the relatedness accurately.

In the early stage, relatedness measurement is based on the low-level visual features such as texture [6, 7], shape [8], and gradient [9]. These visual features are used to represent effective information of an image. Some distance metrics including Chi-Square distance [10], Euclidean distance [11], histogram intersection [12], and EMD distance [13] is used. Overall, these methods ignore the high-level features such as semantic information which can be understood by machine and people easily. These methods are limited to the applications which need semantic level information.

Recently, with the explosion of community contributed multimedia content available online, many social media repositories (e.g., Flickr (http://www.flickr.com), Youtube (http://www.youtube.com), and Zooomr (http://www.zooomr.com)) allow users to upload media data and annotate content with descriptive keywords which are called social tags. We take Flickr, one of the most popular and earliest photo sharing sites, as an example to study the relatedness measurement between images. Flickr provides an open platform for users to publish their personal images freely. The principal purpose of tagging is to make images better accessible to the public. The success of Flickr proves that users are willing to participate in this semantic context through manual annotations [14]. Flickr uses a promising approach for manual metadata generation named “social tagging,” which requires all the users in the social network to label the web resources with their own keywords and share with others. The characteristics of social tags are as follows.

1. Ontology free. The ontology based labeling defines ontology and then let users label the web resources using the semantic markups in the ontology. Social tagging requires all the users in the social network to label the web resources with their own keywords and share with others. Different from ontology based annotation, there is no predefined ontology or taxonomy in social tagging. Thus, the tagging task is more convenient for users.
2. User oriented. The users can annotate images with their favorite tags. The tags of an image are determined by users' cognitive ability. To a same image, users may give different tags. Each image may be with one tag at least, and each tag may appear in many different images.
3. Semantic loss. Irrelevant social tags frequently appear, and users typically will not tag all semantic objects in the image, which is called semantic loss. Polysemy, synonyms, and ambiguity are some drawbacks of social tagging.

**Figure 1:**
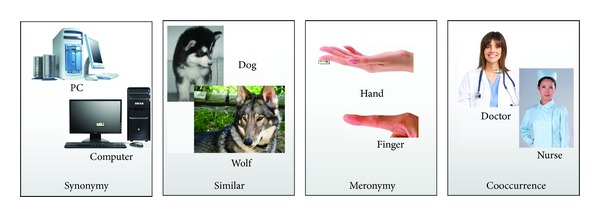
The illustration of four kinds of correlations between concepts.

Based on the above characteristics, we aim at measuring semantic relatedness between images using social tags. It is observed that the correlations between the concepts of images can be divided into four kinds: synonymy, similarity, meronymy, and concurrence, as illustrated in Figure 1. Synonymy means the same object with different names. Similarity denotes that two objects are similar. Meronymy means that two objects follow part-of relation. Concurrence means that two objects appear frequently. Overall, the above four correlations can be summarized as semantic relatedness [15]. Semantic relatedness is a more generic concept than semantic similarity. Similar concepts are usually considered to be related for their likeness (synonymy); dissimilar concepts can also be semantically related such as meronymy or concurrence. In this paper, we focus on measuring semantic relatedness between images.

1. Semantic relatedness follows the cognitive mechanism of people. In [16], the author suggests that the association relation is the basic mechanism of brain. When people know a concept such as “hospital,” she/he may index the related concept such as “doctor” for appropriate understanding of the original concept. Since the goal of relatedness measurement is to facilitate related applications such as searching and recommendation, the proposed method should follow user's cognitive mechanism.
2. Semantic relatedness can be used to organize images based on their associations. In recent literatures, such as Linked Open Data (LOD) [17] and Semantic Link Network (SLN) [18–20], the resources are managed by their semantic relations. The proposed semantic relatedness measures can be used to build semantic links between resources especially images, which can be easily applied in real applications.

The major contributions of this paper are summarized as follows.

1. We propose a framework to measure semantic relatedness between Flickr images using tags. Firstly, the cooccurrence measures are used to compute the relatedness of tags between two images. Secondly, we transform the tags relatedness integration to the assignment in bipartite graph problem, which can find an appropriate matching to the semantic relatedness of images. Finally, a decline factor considering the position information of tags is used in the proposed framework, which reduces the noise and redundancy in the social tags.
2. A real data set including 1000 images from Flickr with ten classes is used in our experiments. Two evaluation methods including clustering and retrieval are performed, which shows that the proposed method can measure the semantic relatedness between Flickr images accurately and robustly.
3. We extend the relatedness measures between concepts to the level of images. Since the association relation is the basic mechanism of brain. The proposed relatedness measurement can facilitate related applications such as searching and recommendation.

The rest of the paper is organized as follows.  Section 2 gives the related work of social tags and image similarity measures. The problem definition is introduced in Section 3.  Section 4 proposes the method for measuring semantic relatedness of images. Experiments are presented in Section 5. Conclusions are made in the last section.

## 2. Related Work

In this section, we give two related aspects of the proposed work. Some researches about social tags are introduced first. Then, we give the related work about image similarity measures.

### 2.1. On Social Tags

In the area about the usage patterns and semantic values of social tags, Golder and Huberman [21] mined usage patterns of social tags based on the delicious (del.icio.us/post) data set. Al-Khalifa and Davis [22] concluded that social tags were semantically richer than automatically extracted keywords. Suchanek et al. [23] used YAGO (http://www.mpi-inf.mpg.de/yago-naga/yago) and WordNet (http://wordnet.princeton.edu) to check the meaning of social tags and concluded that top tags were usually meaningful. Halpin et al. [24] examined why and how the power law distribution of tag usage frequency was formed in a mature social tagging system over time.

Beside research on mining social tags, some researches modeled the network structure of social tags. Cattuto et al. [25] investigated the network features of social tags system, which is seen as a tripartite graph using metrics adapted from classical network measures. Lambiotte and Ausloos [26] described the social tags systems as a tripartite network with users, tags, and annotated items. The proposed tripartite network was projected into the bipartite and unipartite network to discover its structures. In [27], the social tags system was modeled as a tripartite graph which extends the traditional bipartite model of ontologies with a social dimension.

Recently, many researchers investigated the applications of social tags in information retrieval and ranking. In [28], the authors empirically study the potential value of social annotations for web search. Zhou et al. [29] proposed a model using latent dirichlet allocation, which incorporates the topical background of documents and social tags. Xu et al. [30] developed a language model for information retrieval based on metadata property of social tags and their relationships to annotated documents. Bao et al. [31] introduced two ranking methods: SocialSimRank, which ranked pages based on the semantic similarity between tags and pages, and SocialPageRank, which ranked returned pages based on their popularity. Schenkel et al. [32] developed a top-*k* algorithm which ranked search results based on the tags shared by the user who issued the query and the users who annotated the returned documents with the query tags.

### 2.2. On Measuring Images Similarity

Measuring semantic similarity is a basic issue in computer vision field. Usually some low-level visual features are used for similarity measures. For example, shape features, texture features, and gradient features can be extracted from images. Based on the extracted low-level features, distance metrics such as the Euclidean distance, the Chi-Square distance, the histogram intersection, and the EMD distance are used. In this paper, the proposed method addresses the problem by semantic-level features such as social tags.

Different from the methods using low-level features, recently, a number of papers build image representation based on the outputs of concept classifiers [33]. Our observation is that Flickr provides the related social tags by web users, which reflect how people on the internet tend to annotate images. Several previous methods [34] learn object models from internet images. These methods tend to gather training examples using image search results. Besides, their approaches have to alternate between finding good examples and updating object in order to robust against noisy images. On the other hand, some papers [35] use images from Flickr groups other than search engines, which is claimed to be clean enough to produce good classifiers.

## 3. Problem Definition

**Figure 2:**
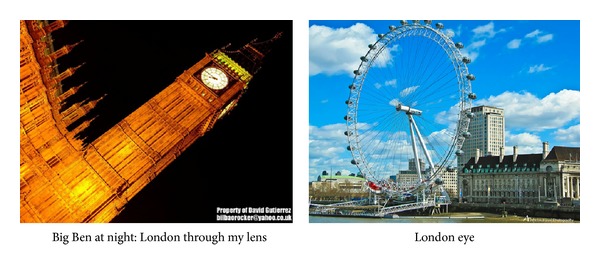
The illustration of a pair of images from Flickr.

In this paper, we study the problem of measuring semantic relatedness between images or videos with manually provided social tags. Here, a social tag refers to some concepts provided by users, which is semantically related to the content of an image or a video. The input of the proposed method is a pair of images or videos with social tags. The goal of the proposed method is to identify the semantic relatedness between two images or videos.  Figure 2 shows the illustration of a pair of images from Flickr with social tags. These two images are about “Big Ben” and “London eye”. These two images may be dissimilar according to the traditional similarity measurement, since they do not share some common low level visual similarity. But, these two images are semantic related since they are both the famous sightseeings of London. In the proposed method, we can compute their semantic relatedness though they may share little similar visual features.

### 3.1. Basic Definitions

We first introduce three important definitions in this paper, the social tags set of an image and the semantic relatedness between two images.

**Definition 1 (social tags set of an image)**

The social tags (denoted by *t*) set of an image *f* (denoted by *s*(*f*)) is a set of tags provided by users of an image:

(1) $$\begin{array}{c} s\left(f\right)=\left\{t_{1},t_{2},\ldots ,t_{\left|s(f)\right|}\right\}. \end{array}$$

For example, in Figure 2, the tags of the right images are “London” and “eye” other than “London eye”. Since Flickr provides the related tags of each image, we just download the tags by Flickr. We do not perform any NLP operations on the tags.

**Definition 2 (semantic relatedness between tags)**

The semantic relatedness between tags (denoted by *sr*⁡(*t*_1_, *t*_2_)) is the expected correlation of a pair of tags *t*_1_ and *t*_2_.

**Definition 3 (semantic relatedness between images)**

The semantic relatedness between images (denoted by *sr*⁡(*f*_1_, *f*_2_)) is the expected correlation of a pair of images *f*_1_ and *f*_2_.

The range of *sr*⁡(*t*_1_, *t*_2_) and *sr*⁡(*f*_1_, *f*_2_) is from 0 to 1. A high value indicates that semantic relatedness between tags or images is more likely to be confidential. Please notice that the definition of *sr*⁡(*f*_1_, *f*_2_) can also be extended to videos with social tags.

### 3.2. Basic Heuristics

Based on common sense and our observations on real data, we have five heuristics that serve as the base of our computation model.

**Heuristic 1**

Usually each tag of an image appears only one time.

Different from writing sentences, users usually annotate an image with different tags. For example, the possibility of using tags “apple apple apple” for an image is very low. Therefore, in this paper, we do not employ any weighting scheme for tags such as tf-idf [36].

**Heuristic 2**

The order of the tags may reflect the correlation against the annotated image.

Different tag reflects the different aspects of an image. According to Heuristic 1, the weight of a tag against the image cannot be obtained. Fortunately, the order of the tags can be gotten since user may provide tags one by one.

**Heuristic 3**

The number of tags of an image may not be relevant to the annotation correctness.

Different users may give different tags about the same image. For example, users may give tags such as “apple iPhone” or “iPhone4 mobile phone” for the same image about iPhone. It is hardly to say which tag is better for annotation though the latter annotation has three tags.

**Heuristic 4**

Usually some tags may be redundant for annotating an image.

Of course, users may give similar tags for an image. For example, the tag “apple iPhone” may be redundant since iPhone is very semantic similar to apple.

**Heuristic 5**

Usually some tags may be noisy for annotating an image.

Users may give inappropriate or even false tags for an image. For example, the tags “iPhone” are false for an image about the iPod.

## 4. Computation Model

In this section, we propose the computation model for measuring semantic relatedness between images. Based on the above five heuristics, the social tags provided by users are used in our computation model. Overall, the proposed computation model is divided into three steps.

1. Tag relatedness computation. In this step, based on Heuristic 1, all of the tag pairs between two images are computed.
2. Semantic relatedness integration. In this step, based on Heuristics 3–5, we measure semantic relatedness between images.
3. Tag order revision. In this step, based on Heuristic 2, the image relatedness on step 2 is revised.

**Figure 3:**
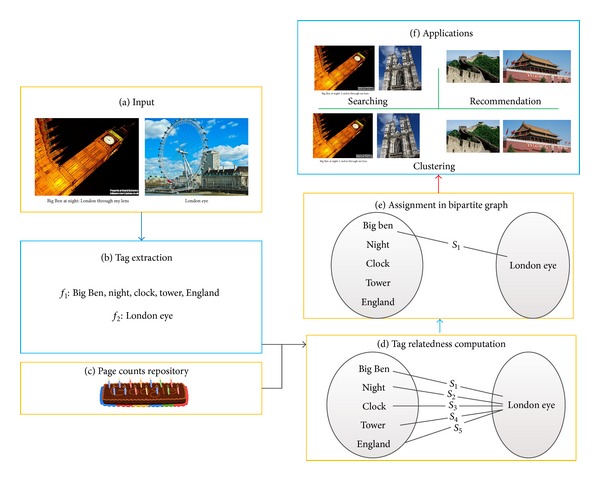
The illustration of the proposed method.

Table 1 shows the variables and parameters used in the following discussion.  Figure 3 illustrates an overview of the proposed computation model.

### 4.1. Tag Relatedness Computation

According to Definition 1, an image can be represented as a set of tags provided by users. As for the semantic relatedness of a pair of images, we can measure the semantic relatedness between tags of these images. For example, two images with tags “apple iPhone” and “iPod Nano”, we can measure the semantic relatedness between these tags. Since the number of each tag is usually one according to Heuristic 1, the semantic relatedness between tags can be computed without considering their weight.

Many different methods of semantic relatedness measures between concepts have been proposed, which can be divided into two aspects [37]: taxonomy-based methods and web-based methods. Taxonomy-based methods use information theory and hierarchical taxonomy, such as WordNet, to measure semantic relatedness. On the contrary, web-based methods use the web as a live and active corpus instead of hierarchical taxonomy.

In the proposed computation model, each tag can be seen as a concept with explicit meaning. Thus, we use some equations based on cooccurrence of two concepts to measure their semantic relatedness. The core idea is that “you shall know a word by the company it keeps” [38]. In this section, four popular cooccurrence measures (i.e., Jaccard, Overlap, Dice, and PMI) are proposed to measure semantic relatedness between tags.

Besides cooccurrence measures, the page counts of each tag from search engine are used. Page counts mean the number of web pages containing the query *q*. For example, the page counts of the query “Obama” in Google (http://www.google.com) are 1,210,000,000 (the data was gotten in the date 9/28/2012). Moreover, page counts for the query “*q* *AND* *p*” can be considered as a measure of cooccurrence of queries *q* and *p*. For the remainder of this paper, we use the notation *N*(*p*) to denote the page counts of the tag *p* in Google. However, the respective page counts for the tag pair *p* and *q* are not enough for measuring semantic relatedness. The page counts for the query “*q* *AND* *p*” should be considered. For example, when we query “Obama” and “United States” in Google, we can find 485,000,000 Web pages; that is, *N*(Obama∩United  States) = 485,000,000. The four cooccurrence measures (i.e., Jaccard, Overlap, Dice, and PMI) between two tags *p* and *q* are as follows:

(2) $$\begin{array}{c} \text{Jaccard}\left(p,q\right)=\frac{N\left(p\cap q\right)}{N\left(p\right)+N\left(q\right)-N\left(p\cap q\right)}, \end{array}$$

*p*∩*q* denotes the conjunction query “*p* *AND* *q*”.

Consider

(3) $$\begin{array}{c} \text{Overlap}\left(p,q\right)=\frac{N\left(p\cap q\right)}{\underset{}{\min}\left(N\left(p\right),N\left(q\right)\right)}, \end{array}$$

min⁡(*N*(*p*), *N*(*q*)) means the lower number of *N*(*p*) or *N*(*q*).

Consider

(4) $$\begin{array}{c} \text{Dice}\left(p,q\right)=\frac{2*N\left(p\cap q\right)}{N\left(p\right)+N\left(q\right)}. \end{array}$$

According to probability and information theory, the mutual information (MI) of two random variables is a quantity that measures the mutual dependence of the two variables. Pointwise mutual information (PMI) is a variant of MI (see (5)):

(5) $$\begin{array}{c} \text{PMI}\left(p,q\right)=\frac{\log\left(\left(N*N\left(p\cap q\right)\right)/\left(N\left(p\right)*N\left(q\right)\right)\right)}{\log N}, \end{array}$$

where *N* is the number of Web pages in the search engine, which is set to *N* = 10^11^ according to the number of indexed pages reported by Google.

Through (2)–(5), we can compute the tag relatedness as follows.

(1) Extracting the tags from two images *f*_1_ and *f*_2_, which are denoted by
  (6) $$\begin{array}{c} s\left(f_{1}\right)=\left\{t_{1},t_{2},\ldots ,t_{\left|s(f_{1})\right|}\right\},\\ s\left(f_{2}\right)=\left\{t_{1},t_{2},\ldots ,t_{\left|s(f_{2})\right|}\right\}. \end{array}$$
(2) Issue the tags from *f*_1_ and *f*_2_ as the query to the web search engine (in this paper, we choose Google for its convenient API (http://developers.google.com)), the page counts can be denoted by
  (7) $$\begin{array}{c} N\left(s\left(f_{1}\right)\right)=\left\{N\left(t_{1}\right),N\left(t_{2}\right),\ldots ,N\left(t_{\left|s(f_{1})\right|}\right)\right\},\\ N\left(s\left(f_{2}\right)\right)=\left\{N\left(t_{1}\right),N\left(t_{2}\right),\ldots ,N\left(t_{\left|s(f_{2})\right|}\right)\right\}. \end{array}$$
(3) Computing the semantic relatedness between each tags pair from *f*_1_ and *f*_2_ by (2)–(5). For example, if we use PMI to compute tag semantic relatedness, the equation can be
  (8) sr⁡(ti,tj)=log⁡((N∗N(ti∩tj))/(N(ti)∗N(tj)))log⁡⁡N,         ti∈s(f1)∧tj∈s(f2).

From the above steps, the tags relatedness can be computed, which is denoted as a triple 〈*t*_*i*_, *t*_*j*_, *sr*⁡(*t*_*i*_, *t*_*j*_)〉. In the next section, we will give the detailed analysis for choosing the best measures from (2)–(5).

Overall, the page counts of each tag should be issued. Then some cooccurrence based measures are used to compute the semantic relatedness between tags. The reasons for using page counts based measures are as follows.

1. Appropriate computation complexity. Since the relatedness between each tag pair of two images should be computed, the proposed method must be with low complexity. Recently, web search engines such as Google provide API for users to index the page counts of each query. The web search engine gives an appropriate interface for the proposed computation model.
2. Explicit semantics. The tag given by users may not be a correct concept in taxonomy. For example, users may give a tag “Bling Bling” for an image about a lovely girl. The word “Bling” cannot be indexed in many taxonomy such as WorldNet. The proposed method uses web search engine as an open intermediate. The explicit semantics of the newly emerge concepts can be gotten by web easily.

### 4.2. Semantic Relatedness Integration

In Section 4.1, we compute the tag pair relatedness of two images. Obviously, the tag pair relatedness of two images *f*_1_ and *f*_2_ can be treated as a bipartite graph, which is denoted by

(9) $$\begin{array}{c} G=\left(V,E\right),\\ V=\left\{f_{1},f_{2}\right\},\\ E=\langle t_{i},t_{j},\mathrm{sr}\left(t_{i},t_{j}\right)\rangle ,\;t_{i}\in s\left(f_{1}\right)\wedge t_{j}\in s\left(f_{2}\right). \end{array}$$

Based on (9), we change the semantic relatedness integration of all tag pairs to the problem—assignment in bipartite graph. We want to assign a best matching of the bipartite graph *G*.

**Figure 4:**
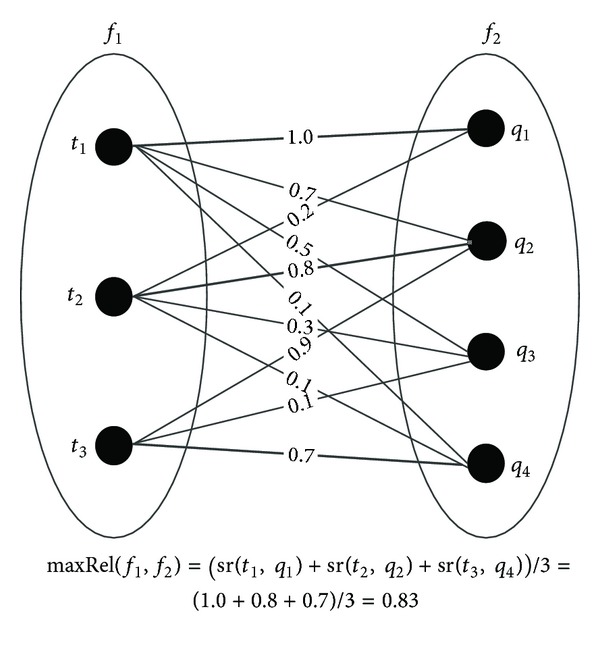
Graphical representation of the assignment in bipartite graphs problem.

A matching is defined as *M*⊆*E* so that no two edges in *M* share a common end vertex. An assignment in a bipartite graph is a matching *M* so that each node of the graph has an incident edge in *M*. Suppose that the set of vertices are partitioned in two sets *f*_1_ and *f*_2_, and that the edges of the graph have an associated weight given by a function *f* : (*f*_1_, *f*_2_) → [0 ⋯ 1]. The function maxRel: (*f*, *f*_1_, *f*_2_) → [0 ⋯ 1] returns the maximum weighted assignment, that is, an assignment so that the average of the weights of the edges is highest.  Figure 4 shows a graphical representation of the semantic relatedness integration, where the bold lines constitute the matching *M*.

Based on the expression of the assignment in bipartite graphs, we have

(10) maxRel(f,f1,f2) ={max⁡⁡∑i∈Ij∈Js(ti,tj)|s(f1)|,|s(f1)|≤|s(f2)|,max⁡⁡∑i∈Ij∈Js(ti,tj)|s(f2)|,|s(f1)|>|s(f2)|,  I=[1⋯|s(f1)|], J=[1⋯|s(f2)|].

Using the assignment in bipartite graphs problem to our context, the variables *f*_1_ and *f*_2_ represent the two images to compute the semantic relatedness. For example, that *f*_1_ and *f*_2_ are composed of the tags *s*(*f*_1_) and *s*(*f*_2_). |*s*(*f*_1_)| > |*s*(*f*_2_)| means that the number of tags in *s*(*f*_2_) is lower than that of *s*(*f*_1_). According to Heuristic 3, we divide the result of the maximization by the lower cardinality of *s*(*f*_1_) or *s*(*f*_2_). In this way, the influence of the number of tags is reduced, and the semantic relatedness of two images is symmetric.

Beside the cardinality of two tags set *s*(*f*_1_) and *s*(*f*_2_), the maxRel function is affected by the relatedness between each pair of tags. According to Heuristics 4 and 5, the redundancy and noise should be avoided. In maxRel function, the one-to-one map is applied to the tags *s*(*f*_1_) and *s*(*f*_2_). Thus, the proposed maxRel function varies with respect to the nature of two images.

Adopting the proposed maxRel function, we are sure to find the global maximum relatedness that can be obtained pairing the elements in the two tags sets. Alternative methods are able to find only the local maximum since they scroll the elements in the first set and, after calculating the relatedness with all the elements in the second set, they select the one with the maximum relatedness. Since every element in one set must be connected, at most, at one element in the other set, such a procedure is able to find only the local maximum since it depends on the order in which the comparisons occur. For example, considering the example in Figure 4,  *t*_1_ will be paired to *q*_1_ (weight = 1.0). But when analyzing *t*_3_, the maximum weight is with *q*_2_ (weight = 0.9). This means that *t*_2_ can no more be paired to *q*_2_ even if the weight is maximum, since this is already matched to *t*_3_. As a consequence, *t*_2_ will be paired to *q*_3_ and the average of the selected weights will be (1.0 + 0.3 + 0.9)/3 = 0.73 which is considerably lower than using MaxRel where the sum of the weights was (1.0 + 0.8 + 0.7)/3 = 0.83.

Overall, the cardinality of two tag sets is used to follow Heuristic 3. The one-to-one map of tags pair is used to follow Heuristics 4 and 5. The MaxRel function is used to match a best semantic relatedness integration of two images.

### 4.3. Tag Order Revision

According to Heuristic 2, the order of tags should be considered to compute the semantic relatedness between two images. Intuitively, the tags appearing in the first position may be more important than the latter tags. Some researches [39] suggest that people used to select popular items as their tags. Meanwhile, the top popular tags are indeed the “meaningful” ones.

In this section, the MaxRel function proposed in Section 4.2 is revised considering the order of tags. For example, the relatedness of tags pair with high position should be enhanced, which is summarized as a constrain schema.

*Schema 1 (tag relatedness declining)*. This schema means that the identical tag pairs of two images *f*_1_ and *f*_2_ should be pruned in MaxRel function. In other words, the semantic relatedness of the same tag of two images is set as 0.

We add a decline factor to the MaxRel function, and the detailed steps are as follows.

(1) According to the MaxRel function in Section 4.2, the best matching tag pairs are selected, which is denoted by
  (11) maxRel(f1,f2)=∑sr⁡(ti,tj),    ti∈s(f1)∧tj∈s(f2).
  Of course, the selected tag pairs are the best matching of the bipartite graph between images *f*_1_ and *f*_2_.
(2) Computing the position information of each tag, which is denoted by Pos(*t*_*i*_):
  (12) $$\begin{array}{c} \text{Pos}\left(t_{i}\right)=\frac{\left|s\left(f\right)\right|+1-i}{\left|s\left(f\right)\right|},\;t_{i}\in s\left(f\right). \end{array}$$
(3) Add the position information of each tag to (11), which can be seen as a decline factor:
  (13) sr⁡(f1,f2)=∑Pos(ti)∗sr⁡(ti,tj)∗Pos(tj),         ti∈s(f1)∧tj∈s(f2).
(4) Of course, similar to MaxRel function, equation should divide the result of the maximization by
  (14) $$\begin{array}{c} \mathrm{sr}\left(f_{1},f_{2}\right)=\frac{\sum \text{Pos}\left(t_{i}\right)*\mathrm{sr}\left(t_{i},t_{j}\right)*\text{Pos}\left(t_{j}\right)}{\sum \text{Pos}\left(t_{i}\right)*\text{Pos}\left(t_{j}\right)}. \end{array}$$

We also consider the example in Figure 4. According to (14), the semantic relatedness is revised as

(15) (1·1.0·1+23·0.8·34+13·0.7·14)  ×(1·1+23·34+13·14)−1=0.92.

Besides adding decline factor to the MaxRel function, we also add a constrain schema: identical tag pruning.

*Schema 2 (identical tag pruning)*. This schema means that the identical tag pairs of two images *f*_1_ and *f*_2_ should be pruned in MaxRel function. In other words, the semantic relatedness of the same tag of two images is set as 0.

The above schema is used to ensure the relatedness measures of two images. If we do not prune the identical tag pairs of two images, the proposed method will be transformed to the similarity measures. For example, the cosine similarity [36] between two tags is to find the number of identical elements of two vectors. The overall algorithm of the proposed computation mode is presented in Algorithm  1.

## 5. Experimental Results 

In this section, we evaluate the results of using the proposed method for relatedness measurement. In Section 5.1, we introduce the data set for the evaluation. In Section 5.2, we determine to use the cooccurrence function for tag relatedness measures. In Sections 5.3 and 5.4, clustering and retrieval are used to evaluate the proposed method.

### 5.1. The Data Sets

We choose Flickr groups as the resources for building data sets. Users on online photo sharing sites like Flickr have organized many millions of photos into hundreds of thousands of semantically themed groups. These groups expose implicit choices that users make about which images are similar. Flickr group membership is usually less noisy than Flickr tags because images are screened by group members. We download 1000 images from ten groups. These ten groups can be divided into two classes. The first class includes five groups, which are car, phone, flower, dog, and boat. The second class consists of another five groups, which are Louis Vuitton, Dior, Gucci, Cartier, and Chanel. Of course, these images are selected by humans, which reduce the noise of the data set. The reason why we choose two classes of groups is that we want to test the accuracy of the proposed method against the semantic relatedness of data set. The semantic relatedness of the second set is higher than the first set since the second class is all about the luxury brands. For example, almost all these brands produce handbags. Thus, if the proposed method can do well in these groups, we may say that it can measure the semantic relatedness between Flickr images accurately and robustly.  Table 2 gives the detailed information of the data set.  Table 3 gives some selected tags from group 2.

### 5.2. Relatedness Function Selection

**Figure 5:**
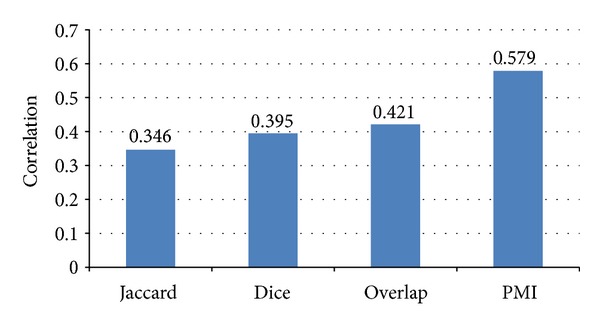
The correlation of four selected functions.

In Section 4.1, four cooccurrence measures (i.e., Jaccard, Overlap, Dice, and PMI) are given for relatedness measures between tags. In [40], Rubenstein and Goodenough proposed a data set containing 28 word pairs rating by a group of 51 human subjects, which is a reliable benchmark for evaluating semantic similarity measures. The higher the correlation coefficient against R-G ratings is, the more accurate the methods for measuring semantic similarity between words are.  Figure 5 gives the correlation coefficient of four functions against R-G test set. From Figure 5, we can say that PMI performs best on relatedness measures for its highest correlation coefficient. Thus, in the later experiments, we select PMI as the relatedness measures between tags.

### 5.3. Evaluation on Image Clustering

**Figure 6:**
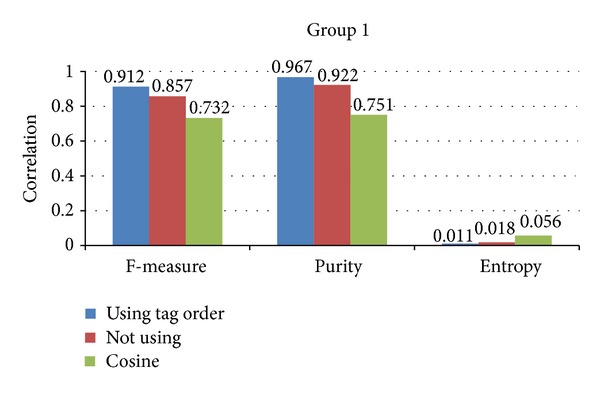
The clustering results of group 1 data sets.

**Figure 7:**
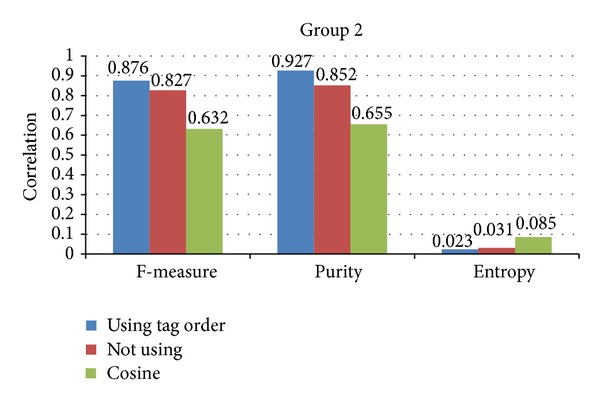
The clustering results of group 2 data sets.

In this section, we evaluate the correctness of using tag order. In Section 4.3, we add the position information of each tag to the semantic relatedness measures. The tags with high position are treated as the major element for sematic relatedness measures. We evaluate the using of tag order by the clustering task. We employ the proposed semantic relatedness of images into *K*-means [41] clustering model. Since the *K*-means model depends on the initial points, we random select core points 100 times. We evaluate the effectiveness of document clustering with three quality measures: *F*-measure, Purity, and Entropy [41]. We treat each cluster as if it were the result of the proposed method and each class as if it were the desired set of images. Generally, we would like to maximize the *F*-measure and Purity and minimize the Entropy of the clusters to achieve a high-quality document clustering. Moreover, we compare the clustering results between the proposed method using tag order or not. Figures 6 and 7 give the clustering results of group 1 and group 2 data sets. From Figures 6 and 7, we can conclude the following.

1. The proposed method performs better than cosine based clustering. This result can be obtained from Figures 6 and 7. The three metrics including *F*-measure, purity, and entropy of the proposed method are better than cosine based clustering. This may be caused by the inherent feature of the proposed method. The proposed method is based on the semantic relatedness other than the cooccurrence of the cosine based clustering. If the tags of two images are not overlapped, the cosine based clustering may be unavailable.
2. The schema on using of tag order is effective. This result can also be obtained from Figures 6 and 7. The three metrics including *F*-measure, purity, and entropy of using tag order are the highest. The position information reflects the importance of each tag. The proposed method emphasizes the tags with high order, which raises the performance on images clustering.
3. The proposed method is robust in different data sets. The proposed method performs well in group 1 and group 2 data set. It is worth noting that the difference between the proposed method and cosine method of group 2 is higher than that of group 1. The reason of that is due to the semantic correlation of group 2 being stronger than group 1. In other words, the performance of the proposed method relies on the semantic correlation of classes in data sets. The stronger the semantic correlation between classes of data, the better the proposed method performance.

### 5.4. Evaluation on Image Searching

In this section, we evaluate the proposed method query-based image searching task. Five queries from group 2 are selected as the test set including “Louis Vuitton,” “Gucci,” “Chanel,” “Cartier,” and “Dior”. These queries are searched in Flickr. The top 50 images are obtained as the data set. Moreover, we remove the queries on the tags of each image. For example, the tag “Cartier” of the top 50 images is removed of the query “Cartier”. The reason for that operation is that the proposed method is based on the semantic relatedness other than cooccurrence. We choose cut-off point precision to evaluate the proposed method on image searching. The cut-off point precision (*P* ^*n*^) means that the percentage of the correct result of the top *n* returned results. We compute the *P* ^1^, *P* ^5^, and *P* ^10^  of the group 2 test set.  Table 4 lists the comparison of the cut-off point precision between the proposed method and Flickr. From the experimental results, we can conclude the following.

1. The proposed method performs better than Flickr. In Table 4, the *P* ^1^, *P* ^5^, and *P* ^10^  of the proposed method are higher than Flickr. The experimental results prove the correctness of the proposed method on image searching task.
2. The proposed method can handle the relatedness searching problem. The proposed method can measure the semantic relatedness of two images robustly and correctly.
3. The proposed method can support the faceted exploration of image search. Faceted exploration of search results is widely used in search interfaces for structured databases. Recently the faceted exploration is also appearing in online search engine in the form of search assistants. The proposed method can measure the semantic relatedness of two images. Given the search queries, we can select the related images for faceted search.

## 6. Conclusions 

This paper mainly discusses the semantic relatedness measures systematically, puts forward a method to measure the semantic relatedness of two images based on their tags, and justifies its validity through the experiments. The major contributions are summarized as follows.

1. We propose a framework to measure semantic relatedness between Flickr images using tags. Firstly, the cooccurrence measures are used to compute the relatedness of tags between two images. Secondly, we transform the tags relatedness integration to the assignment in bipartite graph problem, which can find an appropriate matching to the semantic relatedness of images. Finally, a decline factor considering the position information of tags is used in the proposed framework, which reduces the noise and redundancy in the social tags.
2. A real data set including 1000 images from Flickr with ten classes is used in our experiments. Two evaluation methods including clustering and searching are performed, which shows that the proposed method can measure the semantic relatedness between Flickr images accurately and robustly.
3. We extend the relatedness measures between concepts to the level of images. Since the association relation is the basic mechanism of brain. The proposed relatedness measurement can facilitate related applications such as searching and recommendation.

## Figures and Tables

**Algorithm 1:**
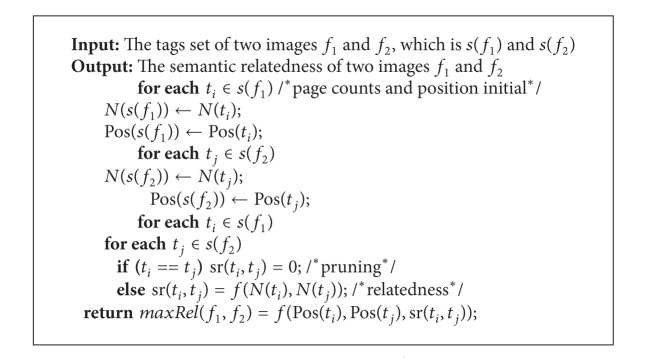
MaxRel.

**Table 1:** The variables and parameters used in the proposed computation model.

Name	Description
*f*	An image
*t*	A tag
*s*(*f*)	Tags set of an image
*sr*⁡(*t*_1_, *t*_2_)	Semantic relatedness of two tags
*sr*⁡(*f*_1_, *f*_2_)	Semantic relatedness of two images
*N*(*t*)	Page counts of a tag
*N*(*s*(*f*))	Set of page counts of an image
pos(*t*)	Position information of a tag

**Table 2:** The detailed information of the data set.

Group 1	Average tags per image	Group 2	Average tags per image
Car	4.4	Louis Vuitton	3.1
Phone	3.5	Dior	3.2
Flower	2.2	Gucci	2.9
Dog	5.6	Cartier	2.8
Boat	3.1	Chanel	2.6

**Table 3:** The selected tags of group 2 from Flickr.

Group 2	Tags	Tags	Tags	Tags	Tags
Louis Vuitton	“Louis Vuitton” “Keepall”	“Louis Vuitton” “Alma”	“Louis Vuitton” “Tivoli”	“Louis Vuitton” “Bolsas”	“LV” “Multicolore”
Dior	“DIOR” “lipstick” “makeup”	“Dior” “Diorskin Nude” “Tan Sun Powder”	“Dior” “Makeup” “Palette”	“Dior” “Addict 2”	“Dior” “Jadore” “Perfume”
Gucci	“Gucci” “Leather Belts”	“Gucci” “Trainers”	“Gucci” “Jolie Leopard” “Orange”	“Replica” “Gucci” “Handbags”	“Gucci” “Cruise”
Cartier	“Cartier” “Pasha” “Chronograph”	“CARTIER” “Love Bracelet”	“Cartier” “Santos Galbee”	“Calibre” “Cartier”	“Cartier Watch” “Tank Francaise”
Chanel	“Chanel” “Coco Noir”	“Chanel” “Chanel Riva” “Chanel nail polish”	“Coco Mademoiselle”	“Chanel” “No 5”	“Chance” “Chanel”

**Table 4:** The comparison of the cut-off point precision between the proposed method and Flickr.

Cut-off point	Louis Vuitton	Gucci	Dior	Chanel	Cartier
*P* ^1^	100%	100%	100%	100%	100%
*P* ^1^ (Flickr)	100%	100%	0	100%	100%
*P* ^5^	100%	100%	100%	100%	100%
*P* ^5^ (Flickr)	80%	60%	60%	60%	80%
*P* ^10^	100%	100%	100%	100%	100%
*P* ^10^ (Flickr)	90%	70%	70%	80%	80%
